# Fecal Microbiota Transplant in Immunotherapy-Resistant Melanoma: What Can We Expect in the Near Future?

**DOI:** 10.7759/cureus.32586

**Published:** 2022-12-16

**Authors:** André Ferreira, Maria Teresa Neves, Ana Baleiras, Mariana Malheiro, Ana Martins

**Affiliations:** 1 Medical Oncology, Hospital de São Francisco Xavier, Lisbon, PRT; 2 Medical Oncology, Hospital CUF (Companhia União Fabril) Tejo, Lisbon, PRT

**Keywords:** immune checkpoint inhibitors, immunotherapy resistance, human gut microbiota, fecal microbiota transplant, melanoma

## Abstract

Melanoma is a malignancy of melanocytes, melanin-producing cells in the basal layer of the epidermis. Despite representing only 1% of skin cancers, melanoma is responsible for over 80% of skin cancer deaths. Treatment with immune checkpoint inhibitors (ICIs) that target the programmed death 1 (PD-1) protein and the cytotoxic T-lymphocyte antigen 4 (CTLA-4) pathways drastically transformed the management of patients with advanced melanoma. Before the introduction of ICIs, the average life expectancy for a patient with advanced melanoma ranged from six to 12 months, and now, this average survival has increased to over six years. However, despite this outstanding clinical success, most patients with advanced melanoma treated with ICIs will experience disease progression, immediately or after an initial response to treatment. Nowadays, some studies have looked at the mechanism behind the resistance to immunotherapy, with the aim of developing new treatments to overcome it. Emerging data suggest that gut microbiota (GM) influences response to immunotherapy. Importantly, unlike tumor genomics, the GM is changeable; thus, modulation of the GM is an attractive approach to overcome immunotherapy resistance. One of these approaches is the fecal microbiota transplant (FMT), which consists of the exchange of manipulated feces from a donor to a recipient who has a disorder related to intestinal dysbiosis to directly change the recipient’s gut microbial composition and confer a health benefit. This review pretends to discuss the clinical benefit of FMT in the treatment of immunotherapy-resistant melanoma and potential adverse effects, including recent and ongoing clinical trials.

## Introduction and background

The gut microbiome contains nearly 100 trillion microorganisms including bacteria, viruses, yeast protozoa, fungi, and archaea. In fact, the microbiome is sometimes referred to as “the last undiscovered human organ” due to its role in maintaining gut mucosal integrity and shaping the development of mucosal and systemic immunity [1]. Moreover, new evidence suggests that it influences oncogenesis and therapeutic outcomes in cancer by regulating local and systemic antitumor immunity [2].

Given that melanoma is one of the most sensitive malignancies to immune modulation, immunotherapy has revolutionized melanoma treatment, both adjuvant and metastatic. Up to now, nearly 20% of metastatic melanoma patients treated with the anti-cytotoxic T-lymphocyte antigen 4 (CTLA-4) monoclonal antibody, ipilimumab, survived at 10 years [3]. In addition, the longest follow-up in metastatic melanoma patients treated with the anti-programmed death 1 (PD-1) monoclonal antibodies, pembrolizumab and nivolumab, reported that 30% of patients were alive at five years [4,5]. This is further improved in melanoma patients who are treated with the combination of ipilimumab plus nivolumab, with overall survival (OS) rate at five years of 52% [6].

However, despite the improvement in long-term outcomes, nearly 40%-50% of tumors are not responsive to single-agent immune checkpoint blockade, and those that do respond can develop long-term resistance [7]. Immunoresistance is clinically defined as primary resistance, if progression occurs within the first six months of therapy, and acquired resistance (secondary), if progression occurs after stable disease after at least six months of immunotherapy [8]. This resistance may be attributed to several factors and can be intrinsic or extrinsic to tumor cells, such as low mutational burden [9], poor intrinsic antigenicity of tumor cells [10], absence of priming by potentially immunogenic pretreatment with chemo- or radiotherapy [11], defective antigen presentation during the primary phase [12], local immunosuppression by extracellular metabolites [13], and functional exhaustion of tumor-infiltrating lymphocytes [14]. Emerging evidence highlights the key role of gut microbiota (GM) in mediating response and toxicity to immunotherapy [15,16]. Melanoma patients treated with antibiotics, alongside immune checkpoint inhibitors (ICIs), had a lower survival rate, and metagenomic analyses of fecal GM showed a compositional difference [17]. Four recent clinical trials, two conducted in Israel, one in the United States, and the other in Canada [18-21], suggest that fecal microbiota transplant (FMT) may restore sensitivity to anti-PD-1 inhibition in immunotherapy-resistant metastatic melanoma.

## Review

Gut microbiota and immunomodulation

The term “gut microbiota” denotes the population of living microorganisms present in the human gastrointestinal (GI) tract. Microbiome denominates the amount of genomic content of these biological agents. Approximately 1000 microorganisms inhabit the human GI tract, which encompasses ~10 times more bacterial cells than the number of human cells and over 100 times the amount of microbiome as the human genome [22,23]. However, a revised estimate published in 2016 showed that the number of bacteria is actually in the same order as the number of human cells [24]. GM offers many benefits to the host, such as helping to maintain the integrity of the mucosal barrier, metabolizing nutrients and drugs, protecting against pathogens, and regulating host immunity.

Strong evidence is emerging to support the effects of GM not only on the pathogenesis of certain malignancies but also on clinical responses to cancer therapies, specifically ICIs [1]. Dysbiosis of the GM is a risk factor for chronic diseases, such as cancer [25]. On the other hand, recent studies have demonstrated that alterations in the GM can increase therapeutic response and reduce immune-related adverse events of ICIs in patients with metastatic melanoma and other types of cancer [26].

Correlation between the gut microbiome and the effectiveness of immunotherapy in melanoma patients

As summarized in Table 1, five studies explored the relationship between baseline composition and diversity of the GM and clinical outcomes of ICIs in metastatic melanoma.

**Table 1:** Clinical trials with an association between gut microbiota and efficacy of ICIs in melanoma patients CTLA-4: Cytotoxic T-lymphocyte-associated protein 4; PD-1: Programmed cell death protein 1; PFS: Progression-free survival; ICIs: Immune checkpoint inhibitors.

Study, Year, Country	Study Population	Findings
Chaputet al., 2017 [27], France	Metastatic melanoma patients (n = 26) who received ipilimumab	Enrichment of *Faecalibacterium*, *Gemmiger*, and *Clostridium XIVa* and impoverishment of *Bacteroides* in anti-CTLA-4 responders (n = 9) comparatively to nonresponders (n = 17).
Frankel et al., 2017 [28], USA	Metastatic melanoma patients (n = 39) who received nivolumab/ipilimumab (n = 24), pembrolizumab (n = 13), nivolumab (n = 1), and ipilimumab (n = 1)	Higher relative abundance of *Bacteroides caccae* in immunotherapy responders (n = 24) comparatively to nonresponders (n = 15). Among ipilimumab (anti-CTLA-4) + nivolumab (anti-PD-1) responders, they noted a higher relative abundance of *Faecalibacterium prausnitzii*, *Bacteroides thetaiotaomicron,* and *Holdemania filiformis*, while pembrolizumab (anti-PD-1) responders verified enrichment of *Dorea formicigenerans.*
Gopalakrishnan et al., 2018 [29], USA	Metastatic melanoma patients (n = 89) who received PD-1 inhibitors	Higher α-diversity, enrichment of *Faecalibacterium prausnitzii*, and impoverishment of *Bacteroides* in anti-PD-1 immunotherapy responders (n = 30) comparatively to nonresponders (n = 59).
Matson et al., 2018 [30], USA	Metastatic melanoma patients (n = 42) who received PD-1 inhibitors (n = 38) and CTLA-4 inhibitors (n = 4)	Enrichment of *Bifidobacterium longum*, *Collinsella aerofaciens*, and *Enterococcus faecium* and impoverishment of *Ruminococcus obeum* and *Roseburia intestinalis* in anti-PD1 immunotherapy responders (n = 16) comparatively to nonresponders (n = 26).
Peters et al., 2019 [31], USA	Metastatic melanoma patients (n = 27) who received PD-1 inhibitors (n = 14), PD-1/CTLA-4 inhibitors (n = 12), and CTLA-4 inhibitors	Higher microbial diversity was associated with longer PFS.

In 2017, Chaputet al. [27] reported that baseline GM enriched with *Faecalibacterium* and other *Firmicutes* is associated with a beneficial clinical response to ipilimumab. In the same year, Frankel et al. [28] reported that while the microbiota differed with the ICI regimen, the enrichment of* Bacteroides caccae* was common in responders treated with any ICI regimen.

In 2018, Gopalakrishnan et al. [29] reported that responders had a higher relative abundance of *Faecalibacterium prausnitzii *than nonresponders. Also in 2018, Matson et al. [30] found that the enrichment of a group of eight species driven by *Bifidobacterium longum* was correlated with responsiveness to PD-1 therapy.

In the following year, Peters et al. [31] reported that higher microbial community richness was associated with longer progression-free survival (PFS) (p < 0.05). They found that the abundance of *F. prausnitzii *and other protective species was associated with longer PFS, while an abundance of *Bacteroides *species was related to shorter PFS.

Modulation of the gut microbiome with FMT in advanced melanoma patients

The therapeutic potential to improve patient outcomes by modulating the GM is an exciting one [1]. GM can be manipulated through different strategies such as diet or probiotic supplements. However, the most well-established method of GM modulation is through FMT, whereby a donor microbiome is introduced into another individual through an acid-resistant capsule or endoscopically [32].

Four recent studies analyzed the impact of FMT on the efficacy of ICIs (Table 2) and reported that FMT from donors who had been previously treated with anti-PD-1 monotherapy for metastatic melanoma achieved a complete or partial response, while patients with anti-PD-1 refractory metastatic melanoma demonstrated a clinical response to ICIs in a subgroup of patients when they were treated with the association of FMT with anti-PD-1 immunotherapy.

**Table 2:** Clinical trials that evaluated the efficacy of FMT in patients with anti-PD-1 refractory metastatic melanoma PD-1: Programmed cell death protein 1; FMT: Fecal microbiota transplant.

Study, Year, Country	Study Population	Results
Baruch* *et al., 2021 [18], Israel	Metastatic melanoma patients (n = 10) who progressed on anti-PD-1 therapy received FMT from two donors who had previously been treated with nivolumab for metastatic melanoma and achieved complete response for at least one year.	Three patients responded (one with a complete response and two with a partial response). The treatment was well-tolerated with no severe adverse events related to FMT and no grade ≥2 immune-related adverse events.
Davar et al., 2021 [19], USA	Metastatic melanoma patients (n = 15) with primary refractory to anti-PD-1 therapy received FMT from seven donors who achieved complete response (n = 4) or partial response (n=3) to pembrolizumab.	Six patients responded (three with partial response and three with stable disease).
Youngster et al., 2019 [20], Israel	Metastatic melanoma patients (n = 5) who progressed on anti-PD-1 therapy received FMT from two donors who achieved a durable complete response to treatment.	Three patients had a partial or complete response to treatment post-FMT. FMT in metastatic melanoma patients seems to be safe and may alter the recipient gut microbiota to resemble that of a responder donor.
Maleki et al., 2020 [21], Canada	Metastatic melanoma patients (n = 2) who progressed on anti-PD-1 therapy received FMT from two donors.	FMT combined with anti-PD1 therapy in patients with advanced melanoma appears to be safe. A measurable immune response was observed one week after FMT in both patients. One patient experienced several grade 2 toxicities with stabilization of a large cutaneous lesion.

Baruch et al. [18] examined 10 patients with metastatic melanoma, who were resistant to ICIs, which is defined as the inability to achieve a durable response to anti-PD-1 treatment. In their study, FMT products were extracted from two donors who had achieved a complete response to nivolumab for metastatic melanoma for at least one year. Before the FMT administration, patients underwent antibiotic therapy with vancomycin and neomycin for three days to deplete native microbiota. FMT was then performed via both colonoscopy and administration of oral stool capsules followed by re-introduction of anti-PD-1 therapy (nivolumab). Three patients (30%) overcame therapeutic resistance after FMT, one with a complete response and two with a partial response. They concluded that FMT in addition to the re-introduction of immunotherapy was safe, feasible, and potentially effective.

A similar study with five patients was performed by Bibbò et al. [33], and three of them (60%) had a partial or complete response to immunotherapy after FMT. They also found a post-FMT compositional dissimilarity with an increased abundance of *Paraprevotellaceae*, previously associated with responsiveness to treatment, and a significant decrease in β-proteobacteria, previously associated with resistance to treatment.

Davar et al. [19] investigated the effect of FMT in 15 melanoma patients with PD-1 refractory metastatic melanoma. FMT products were derived from seven donors who had a partial or complete response to pembrolizumab. After FMT, six patients (40%) showed a response to anti-PD-1 therapy, three with stable disease and three with partial response. Responders also showed more diversity in the gut microbiome, more CD8+ T-cell activation, and less interleukin-8-expressing myeloid cell, which are related to immunotherapy resistance.

Finally, Sharma et al. [34] evaluated two patients with recurrent metastatic melanoma that received FMT from two different donors. One patient presented with stabilization of a solitary large cutaneous lesion, while there was no description of the tumor response of the other patient. The CD39+ CD8+ T-cell population increased, and the PD1+, CD38+, and CD8+ dysfunctional T-cell levels decreased after the treatment with FMT plus anti-PD1 therapy, in both patients.

Future trials

Based on these promising results, several clinical trials assessing FMT in melanoma patients are still ongoing (clinicaltrial.gov).

A clinical trial (NCT05251389) is currently investigating if the FMT of either responder or nonresponder patients can convert the response to immunotherapy in ICI refractory metastatic melanoma patients. The primary outcome is efficacy, defined as a clinical benefit (stable disease, partial response, or complete response) at 12 weeks after FMT.

The PICASSO trial (NCT04988841) is a French phase 2 prospective randomized trial that aims to assess the tolerance and clinical benefit of FMT in patients with melanoma in addition to the usual treatment with immunotherapy combining ipilimumab and nivolumab. It is the first randomized trial of FMT in patients with unresectable or metastatic melanoma. The investigators will compare fecal transplantation using MaaT013 (a donor-derived, standardized, high-richness, high-diversity microbiome ecosystem therapy, containing a group of bacterial species known to produce anti-inflammatory short-chain fatty acids) with a placebo in 60 patients. The primary endpoint is the safety of a 23-week treatment with MaaT013 in combination with nivolumab plus ipilimumab.

The IRMI-FMT trial (NCT04577729), an Austrian study, claims to investigate the effect of FMT and ICI re-challenge on PFS and tumor in prior ICI refractory patients using donor stool of former malignant melanoma patients, who have been in remission due to ICI treatment for at least one year. They will compare autologous FMT with allogenic FMT from prior melanoma patients in remission for at least one year after ICI treatment, concurrently with ICI re-challenge. The primary endpoint is PFS evaluated by immune-RECIST (response evaluation criteria in solid tumors) three months after ICI therapy following FMT.

Limitations of FMT

Despite microbiota modulation by FMT in association with the re-introduction of ICIs might have a promising therapeutic potential, FMT use in cancer immunotherapy resistance has several key limitations.

Although favorable and unfavorable species of bacteria have been identified, the composition of an “ideal GM” remains unknown. The ideal GM differs from person to person and may be dependent on several modifiable and non-modifiable factors, concluding that it is unlikely to be a one-size-fits-all GM. Donor selection represents a fundamental challenge in view of the implementation of FMT programs worldwide [33]. The appropriate microbiota composition for donors is not yet established at present. It is also still unclear why some patients responded to the combination of FMT and ICI, while others did not. The reason could be due to the presence of additional immune checkpoints, such as the lymphocyte activation gene 3 (LAG-3), T-cell immunoglobulin and mucin domain 3 (TIM-3), and T-cell immunoreceptor with Ig and ITIM domains (TIGIT), or lack of proper antigen presentation machinery in tumor cells [34]. As a result, there are currently no available screening tools or prognostic factors that may be used to stratify potential recipient patients for FMT-ICI treatment [35].

Another limitation is related to the significant infectious risk that entails the transfer of fecal content from one human to another. For this reason, FMT is not an approved treatment by both the Food and Drug Administration or European Medicines Agency, even for recurrent *Clostridium difficile* colitis. To minimize FMT-associated risks, there are safety screening recommendations from regulatory agencies and clinical guidelines for potential donors [36,37]. Given that those safety restrictions demand a large pre-screening donor pool, it is more viable to recruit potential donors among the general healthy population than the smaller donor pool of cancer patients who responded to ICIs. However, in the four clinical trials previously mentioned, the donors were metastatic melanoma patients who responded to ICIs.

## Conclusions

This review analyzes the evidence on the role of FMT in immunotherapy-resistant melanoma. Colitis, one of the most common adverse effects of immunotherapy, is directly associated with particular microbiological signatures. Thus, the modulation of the intestinal microbiota constitutes a promising tool in oncologic treatment. Several tools have been tested with promising results, from traditional probiotics to innovative and intriguing FMT. For these reasons, there is a burgeoning optimism regarding the modulation of the microbiota in melanoma patients treated with immunotherapy. However, the available studies are still preliminary, with a small sample of patients and some limitations, such as the risk of transmission of bacteria or pathogens, the sample variability in terms of the type of patients enrolled, the immunotherapy administered, and the modulation methodology of the microbiota used.

Therefore, more preclinical and clinical trials are necessary to confirm the safety and effectiveness of GM modulation in immunotherapy treatment in order to implement and standardize it. It is especially essential to accurately recognize the bacterial species that predispose to the development of a lower response to immunotherapy or adverse events and specific probiotics, which can selectively be administered to improve clinical responses. In conclusion, the modulation of the GM, particularly with FMT, emerges as a fascinating therapeutic tool in the treatment of patients with anti-PD-1 refractory metastatic melanoma, and this greater propagation of knowledge about the relationship between the GM and the immune system lets us hope for the best in the near future.
